# Supplementary material for: A Global Screen for Assembly State Changes of the Mitotic Proteome by SEC-SWATH-MS
Source: Cell Syst. 2020 Feb 26;10(2):133–155.e6. doi: 10.1016/j.cels.2020.01.001 (PMC7042714; doi:10.1016/j.cels.2020.01.001)

Q9NXA8 | SIR5_HUMAN | SIRT5 SIR2L5
Monomer MW [kDa]: 33.881 Monomer expected elution fraction: 50

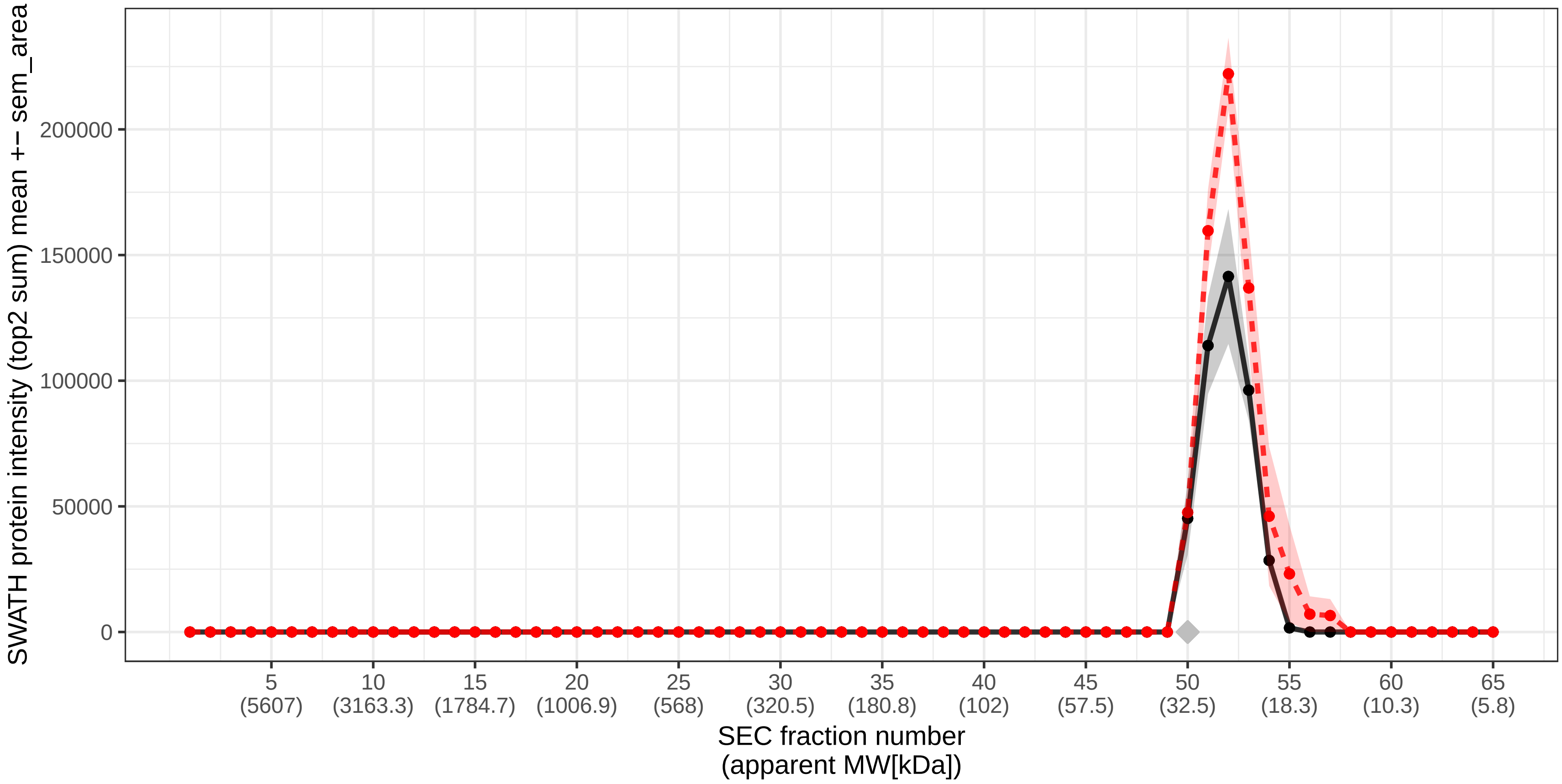

Supplement: Data S1. SEC-SWATH-MS Protein Chromatograms, Related to Figure 1 [file mmc6.zip › SECchrom_Q9NXA8_SIR5_HUMAN_SIRT5_SIR2L5.pdf]
